# Expression Characteristics of 3-Marker Panel (PAX2, PTEN, and β-Catenin) in Benign Interval and Secretory Endometrium and Secretory Endometrial Precancer

**DOI:** 10.3390/cancers17091495

**Published:** 2025-04-29

**Authors:** Shuang Niu, Kyle Molberg, Jackson Chen, Lesley Conrad, Elena Lucas, Hao Chen

**Affiliations:** 1 Department of Pathology, UT Southwestern Medical Center, Dallas, TX 75390, USA; shuang.niu@utsouthwestern.edu (S.N.); kyle.molberg@utsouthwestern.edu (K.M.); 2 Department of Pathology, Parkland Hospital, Dallas, TX 75235, USA; 3 Coppell High School, Coppell, TX 75019, USA; jackson.chen0224@gmail.com; 4 Department of Obstetrics and Gynecology, UT Southwestern Medical Center, Dallas, TX 75390, USA; lesley.conrad@utsouthwestern.edu

**Keywords:** secretory atypical hyperplasia/endometrioid intraepithelial neoplasia, PAX2, PTEN, β-catenin, immunohistochemistry

## Abstract

**Background/Objectives**: Despite advancements in treatment options, endometrial cancer remains a significant threat to women, underscoring the importance of early detection of atypical hyperplasia/endometrioid intraepithelial neoplasia (AH/EIN), the widely accepted histological precursor to endometrial carcinoma. Even with refinements in morphological criteria for the diagnosis of AH/EIN, accurate diagnosis remains challenging for pathologists, particularly in cases with secretory changes. Morphological alterations resulting from secretory-related changes further complicate the application of diagnostic criteria, emphasizing the need for reliable biomarkers. Previous studies have demonstrated that a panel consisting of three immunohistochemical markers, PAX2, PTEN, and β-catenin, can be effectively utilized for the identification of AH/EIN in various non-secretory scenarios. **Methods**: In this study, a total of 69 AH/EIN within secretory endometrium were analyzed using this panel. Benign interval endometrium (*n* = 57) and secretory phase endometrium (*n* = 71) were also analyzed for PAX2, PTEN, and β-catenin expression as controls. **Results**: The 3-marker panel successfully identified 93% of secretory AH/EIN, comparable to its performance in identifying non-secretory bona fide AH/EIN (92.8%) and AH/EIN within endometrial polyps (92.4%). Of note, β-catenin expression in benign interval endometrium commonly displayed weak nuclear staining (67%), which could pose a diagnostic pitfall when using the 3-marker panel. Overall, the results demonstrate the diagnostic utility of the 3-marker panel in clinical practice in identifying AH/EIN within challenging secretory phase endometrium cases. **Conclusions**: Combined with previous research highlighting its effectiveness in other challenging contexts—such as AH/EIN in polyps, small-sized EIN (subdiagnostic EIN), and progestin-treated EIN—this study provides strong evidence supporting the panel’s broad applicability in resolving major diagnostic challenges related to the precise diagnosis of AH/EIN.

## Simple Summary

Endometrial cancer is a serious concern, and detecting its precursor—atypical hyperplasia/endometrioid intraepithelial neoplasia (AH/EIN)—is crucial for prevention. However, diagnosing these precancers can be challenging, especially when they undergo secretory changes (also known as secretory AH/EIN). This study evaluated a three-marker panel (PAX2, PTEN, and β-catenin) to improve the accuracy of identifying secretory AH/EIN. We analyzed 69 secretory AH/EIN cases and compared them to 57 benign interval endometrium and 71 secretory phase endometrium samples. The panel successfully identified 93% of secretory AH/EIN cases, performing comparably to its effectiveness in non-secretory cases. However, weak β-catenin staining in some benign interval endometrium samples could lead to false-positive interpretations. Overall, the findings confirm that this marker panel is a valuable tool for diagnosing AH/EIN, even in complex cases, and supports its broader clinical use, including in polyps and hormone-treated conditions.

## 1. Introduction

Endometrial cancer is the most common gynecological cancer and the fourth most common type of female cancer overall in the United States [1,2]. Over the past decades, its incidence has been steadily rising and remains a significant threat to women despite advances in medicine [3]. Therefore, it is of utmost importance to accurately detect endometrial cancer at its early stage or its precancerous form.

Atypical Hyperplasia/Endometrioid Intraepithelial Neoplasia (AH/EIN) is widely recognized as the precursor to endometrioid carcinoma [4]. According to the WHO 2020 [4], the morphological diagnostic criteria of AH/EIN include the following: (1) Glandular crowding—gland to stroma ratio >1; (2) Cytological atypia—distinctiveness in nuclear and/or cytoplasmic features from the background endometrium. Common features of nuclear atypia include various combinations of loss of polarity, irregular nuclear shape and contours, vesicular chromatin, hyperchromasia, and prominent nucleoli; (3) Sufficient size of the lesion to exclude artifactual crowding; (4) Exclusion of carcinoma or mimics such as endometrial polyps. However, the diagnosis of AH/EIN continues to remain challenging and subjective in the following scenarios: AH/EIN within endometrial polyps (aka. AH/EIN in polyps), small-sized AH/EIN (subdiagnostic AH/EIN), progestin-treated AH/EIN, and AH/EIN with secretory changes (aka. secretory AH/EIN) [5]. Secretory AH/EIN poses a particular difficulty in diagnosis because: (1) glandular crowding is also a feature of secretory endometrium, and (2) nuclear atypia is often less prominent or absent in secretory AH/EIN due to the influence of high circulating levels of progesterone.

Genomic studies in recent years have significantly increased our knowledge of the underlying molecular mechanisms of endometrial carcinoma and AH/EIN. The Cancer Genome Atlas (TCGA) data have shown that, in endometrial cancer, the two most frequently disrupted intracellular signaling pathways include the PI3K-PTEN-AKT-mTOR and the canonical WNT-β-catenin pathway [6]. Some frequently mutated genes that may contribute to these pathway interruptions include PTEN, CTNNB1, ARID1A, and KRAS. These genes are also frequently mutated in AH/EIN, making them strong candidates for immunohistochemical biomarkers in diagnosing AH/EIN.

PTEN dephosphorylates PIP3 to PIP2, thereby inhibiting pathway activation. Loss of PTEN expression disrupts this regulatory checkpoint, resulting in uncontrolled cell growth. β-catenin, encoded by the *CTNNB1* gene, plays a central role in the canonical WNT/β-catenin signaling pathway, which regulates cell proliferation, differentiation, and carcinogenesis [7,8]. Mutations in exon 3 of *CTNNB1*, a known mutational hotspot, affect phosphorylation sites that normally tag β-catenin for degradation. These mutations prevent its degradation, leading to nuclear accumulation and overexpression, which may contribute to tumorigenesis. Although PAX2 is not among the commonly mutated genes reported in TCGA data, aberrant loss of nuclear PAX2 expression has been observed in approximately 80% of AH/EIN [9]. The underlying mechanism for this loss remains unclear, though epigenetic silencing has been proposed [10,11]. Notably, PAX2 loss occurs early in the development of AH/EIN [6,9,12,13,14,15,16,17,18,19], making it a promising early diagnostic marker. PTEN is a tumor suppressor and lipid phosphatase that regulates the PI3K-AKT-mTOR pathway, a critical signaling cascade involved in cell survival, growth, and proliferation [20].

Previous studies have narrowed potential markers into a 3-marker panel (PAX2, PTEN, and β-catenin), demonstrating excellent accuracy in bona fide AH/EIN [9] and challenging scenarios, including AH/EIN in polyps and subdiagnostic AH/EIN [21,22]. A longitudinal investigation of PAX2 and PTEN expression patterns in serial biopsies from women treated with progestin revealed that pretreatment AH/EIN expression patterns were consistently recapitulated in post-treatment AH/EIN. These findings suggest the potential utility of this panel in diagnosing progestin-treated AH/EIN [23]. Additionally, prior research has examined the expression patterns of these markers in large cohorts of benign endometrium, including proliferative endometrium and endometrial polyps [9,21], further supporting the high specificity of this panel in detecting AH/EIN.

The aims of this study were to investigate: (1) The expression characteristics of the 3-marker panel in interval endometrium and secretory endometrium to refine its interpretation criteria; (2) The diagnostic sensitivity and specificity of the 3-marker panel in identifying secretory AH/EIN.

## 2. Materials and Methods

### 2.1. Case Selection

Following approval from the Institutional Review Board of the University of Texas Southwestern Medical Center, 69 cases of secretory AH/EIN were identified for analysis, along with 128 cases of benign endometrium serving as controls, including 57 interval and 71 secretory phase endometrium cases. All cases were de-identified and assigned numerical labels. The criteria applied for diagnosing secretory AH/EIN include cytologic distinctiveness from the background endometrium (with or without bona fide nuclear atypia), morphologic demarcation of the focus, and sufficient lesion size. A minimum size of approximately 1 mm was used in this study to confidently exclude mimics, such as glands naturally lagging in secretory development, artifactual crowding, or carcinoma. The interval phase of the endometrium (days 15–16 of the endometrial cycle) is characterized by less than 50% of glandular cells containing continuous and subnuclear vacuoles. The diagnoses were verified by three gynecologic pathologists (S.N., E.L., and H.C.).

### 2.2. Immunohistochemistry

Immunohistochemistry for PAX2, PTEN, and β-catenin was performed in the clinical laboratory on a DAKO Autostainer Link 48 instrument, following the staining protocols validated for clinical testing. The following primary antibodies were used: PAX2 (Prediluted, clone EP235, #BSB2567, Cancer Diagnostics, Durham, NC, USA), PTEN (Prediluted, clone 6H2.1, #PM278AA, BioCare, Pacheco, CA, USA), and β-catenin (Prediluted, clone β-catenin-1, #IR70261-2, Agilent, Santa Clara, CA, USA). Antigen retrieval was performed in low pH (6.0) for β-catenin and high pH (9.0) Tris/EDTA solution for PAX2 and PTEN at 97 C for 20 min. FLEX peroxidase block was performed for 10 min for β-catenin and 5 min for PAX2 and PTEN. Primary antibody incubation time was 40 min for PAX2 and PTEN, and 20 min for β-catenin. In all cases, the enzymatic conversion of the 3,3′-diaminobenzidine tetrahydrochloride chromogen was performed for 10 min at room temperature, and hematoxylin counterstaining was then performed.

### 2.3. Interpretation of 3-Marker Panel

The interpretation of IHC slides was performed by three gynecologic pathologists (S.N., E.L., and H.C.). Nuclear atypia was analyzed according to the previously described specifications. The interpretation of β-catenin, PAX2, and PTEN staining was performed following previously published criteria with slight modifications [5]. Briefly, the absence of staining in more than 10% of the specimen was considered loss of expression (aberrant expression) for PAX2 and PTEN. Strong, unequivocal nuclear β-catenin staining in any proportion of the glands was interpreted as aberrant expression. The assessment of the 3-marker panel was based exclusively on the glandular component.

### 2.4. Statistical Analysis

Marker expression differences between groups were evaluated using either the two-tailed chi-square (χ^2^) test or Fisher’s exact test, depending on sample size and distribution. A *p*-value of less than 0.05 was considered statistically significant. The Phi coefficient (Φ) was used to measure the strength and direction of associations between individual markers, indicating the degree of co-occurrence beyond chance. Φ values range from −1 to 1, with 1 indicating perfect positive correlation, 0 no correlation, and −1 perfect negative correlation. Values greater than 0.4 or less than −0.4 were interpreted as strong associations, while values between −0.2 and 0.2 suggested weak or no association. All analyses were conducted using SPSS version 27.0 (IBM Corp., Armonk, NY, USA).

## 3. Results

### 3.1. Expression Characteristics of PAX2, PTEN, and β-Catenin in Benign Interval and Secretory Endometrium

**Figure 1:**
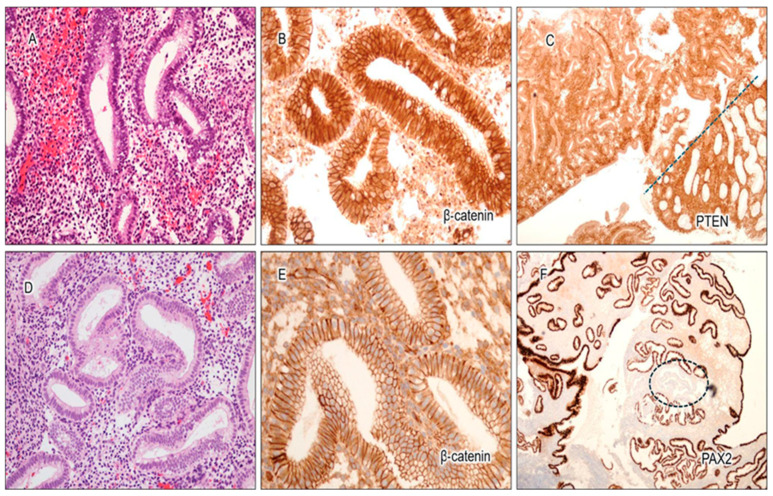
Expression characteristics of 3-marker panel in benign endometrial glands in interval and/or secretory phase endometrium. (**A**) Representative image of Hematoxylin & Eosin (H&E, 10X) staining of interval endometrium. (**B**) Weak nuclear staining can be frequently seen only in interval endometrium (20X). (**C**) Occasional focal/scattered PTEN loss can be observed in benign interval and secretory phase endometrium (4X). Loss of PTEN expression is located to the right of the blue line, while intact PTEN expression is found to the left. (**D**) Representative image of secretory phase endometrium(H&E, 10X). (**E**) Characteristic membrane staining of β-catenin in benign secretory endometrium (20X). (**F**) Occasional focal/scattered PAX2 loss is not uncommon in benign interval/secretory phase endometrium. PAX2 expression loss is present inside the blue circle (4X).

Sporadic focal loss of PAX2 and PTEN was not uncommon in benign interval and secretory endometrium (Figure 1). Among the 128 benign cases analyzed (*n* = 57 interval, *n* = 71 secretory), PAX2 and PTEN were fully intact in 91% (*n* = 117) and 71% (*n* = 91) of cases, respectively. Cases with PAX2 and PTEN loss were further categorized into the following groups: <1%, 1–5%, 6–10%, and >10% loss. Regarding PAX2 expression, 8% of cases showed less than 1% loss, 1% had between 1 and 5% loss, and none had greater loss of expression. For PTEN expression, 16% of cases showed less than 1% loss, 12% had between 1 and 5% loss, less than 1% had between 6 and 10% loss, and no other cases exhibited greater loss of expression. The distribution of cases with PAX2 or PTEN loss is illustrated in Figure 1C,F.

**Figure 2:**
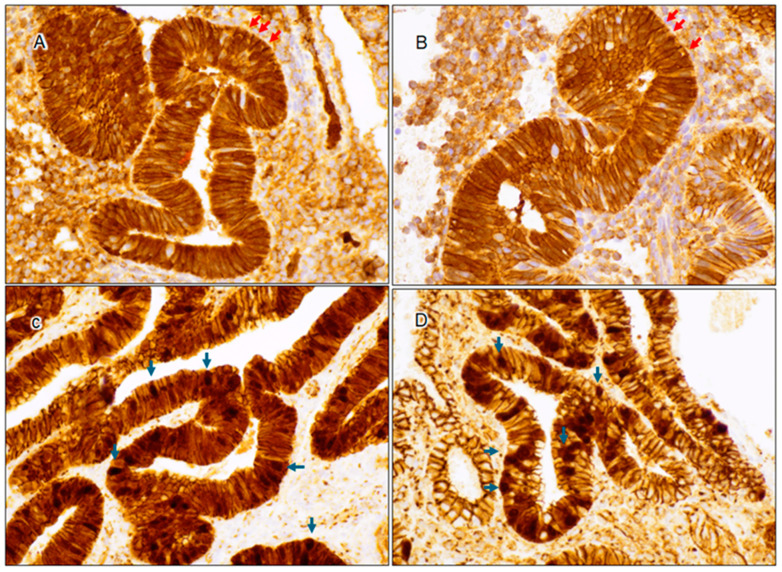
Comparison of β-catenin expression: weak nuclear expression in interval endometrium (**A**,**B**) (40X) versus strong nuclear expression in AH/EIN (**C**,**D**) (40X). Red arrows indicate weak β-catenin nuclear expression in interval endometrium, while blue arrows indicate bona fide strong β-catenin nuclear expression.

**Figure 3:**
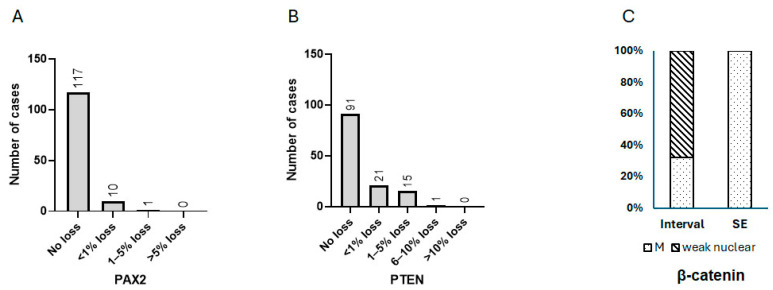
Expression characteristics of 3-marker panel in interval/secretory endometrium. (**A**) Distribution of benign interval/secretory endometrium exhibiting various levels of PAX2 loss. (**B**) Distribution of benign interval/secretory endometrium exhibiting various levels of PTEN loss. (**C**) Percentage of interval/secretory phase endometrium exhibiting weak β-catenin staining. M: Membranous.

The expression pattern of β-catenin was of particular interest. Weak nuclear staining was observed in 67% (*n* = 38) of the interval cases (Figure 1B, Figure 2A,B and Figure 3C), compared to strong nuclear staining in AH/EIN (Figure 2C,D). This weak nuclear staining was absent in 100% of the secretory endometrium cases. Not one of the 128 cases showed strong, unequivocal nuclear staining characteristic of AH/EIN.

Furthermore, out of the 57 interval cases, fully intact expression of PAX2 and PTEN was found in 88% and 58% of cases, respectively. For PAX2, the remaining 12% of cases had less than 1% loss of expression. For PTEN, 26% of cases had less than 1% loss, 14% of cases had between 1 and 5% loss, and 2% of cases had 6–10% loss. Out of the 71 secretory phase cases, a completely normal expression of PAX2, PTEN, and β-catenin was observed in 94%, 82%, and 100% of cases, respectively. For PAX2, 4% of cases had <1% loss, 1% of cases had 1–5% loss, and no cases had more than this amount. For PTEN, 8% of cases had <1% loss, 10% of cases showed 1–5% loss, and no other cases had more than this amount. The expression characteristics of the three markers in interval/secretory endometrium are illustrated in Figure 3.

### 3.2. Cytological Analysis and Analysis of PAX2, PTEN, and β-Catenin in Secretory AH/EIN

**Figure 4:**
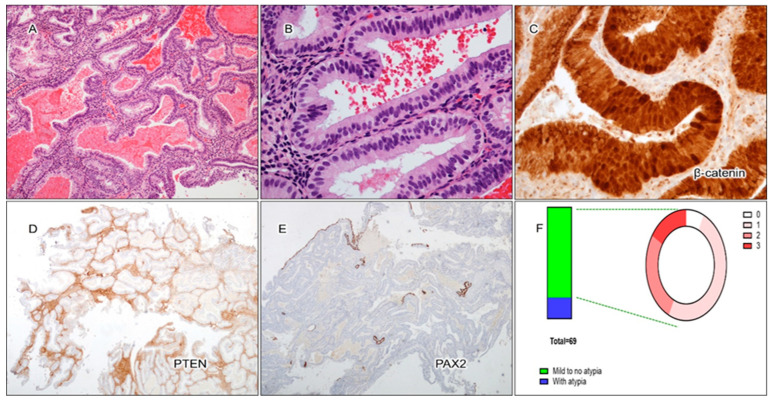
Example of aberrant 3-marker expression in AH/EIN with secretory changes (secretory AH/EIN). (**A**) Low power (H&E staining, 4X). (**B**) High power (H&E staining, 40X). No nuclear atypia and lack of mitosis. (**C**) Aberrant nuclear staining of β-catenin (40X). (**D**) Loss of PTEN expression (4X). (**E**) Loss of PAX2 expression (4X). (**F**) Performance of 3-marker panel in secretory AH/EIN with mild to no nuclear atypia.

**Figure 5:**
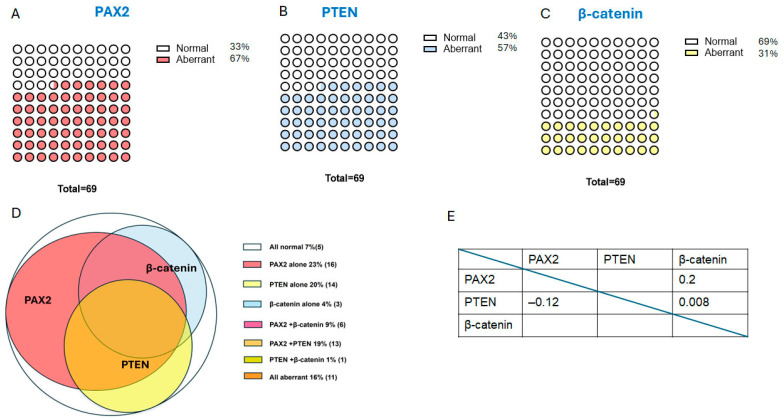
Distribution of marker aberrancy in secretory AH/EIN. (**A**–**C**) Parts-of-whole representations for individual markers in secretory AH/EIN. Graphs represent an idealized set of 100 patients with aberrancy (filled circles) of each of the 3 markers based on the analysis of 69 patients. (**D**) Venn diagram demonstrating distribution of marker aberrancy among secretory AH/EIN cases. (**E**) Associations among all pairwise combinations of markers. Positive associations are positioned on the upper right, negative associations on the lower left. The numbers inside the cells represent the Φ values.

Out of the 69 cases of secretory AH/EIN, the majority of cases (81%) displayed minimal to no nuclear atypia (Figure 4A,B,F). This was significantly different from non-secretory EIN cases, where nuclear atypia was present in the majority of cases. Aberrant expression of PAX2, PTEN, and β-catenin was observed in secretory AH/EIN in 67%, 57%, and 31% of cases, respectively. At least one of these markers showed abnormal expression in 93% of the cases of AH/EIN during the secretory phase. A total of 16% of the cases displayed aberrant expression of all three markers, 29% of cases displayed two out of three markers as aberrant, and 47% of cases displayed only one abnormal marker. All pairwise comparisons showed no or only a weak association among the individual markers. A more detailed breakdown of the pattern of expression can be found in Figure 5 and Table 1.

There was no significant difference between the expression patterns of the markers in cases with mild to no atypia compared to cases with nuclear atypia. For secretory EIN cases with minimal to no nuclear atypia, abnormal expression of β-catenin (Figure 4C), PTEN (Figure 4D), and PAX2 (Figure 4E) was observed in 32%, 54%, and 66% of cases, respectively. A total of 16% of cases had all three markers abnormal, 27% of cases displayed two out of three markers as aberrant, and 93% of all cases displayed at least one marker as abnormal. For secretory cases with nuclear atypia, abnormal expression of PAX2, PTEN, and β-catenin was observed in 69%, 69%, and 23% of cases, respectively. A total of 15% of all cases had all three markers abnormal, 38% of cases displayed two out of three markers as aberrant, and 92% of all cases displayed at least one marker as abnormal. The aberrant patterns of expression for the three markers in secretory AH/EIN are summarized in Table 1.

**Figure 6:**
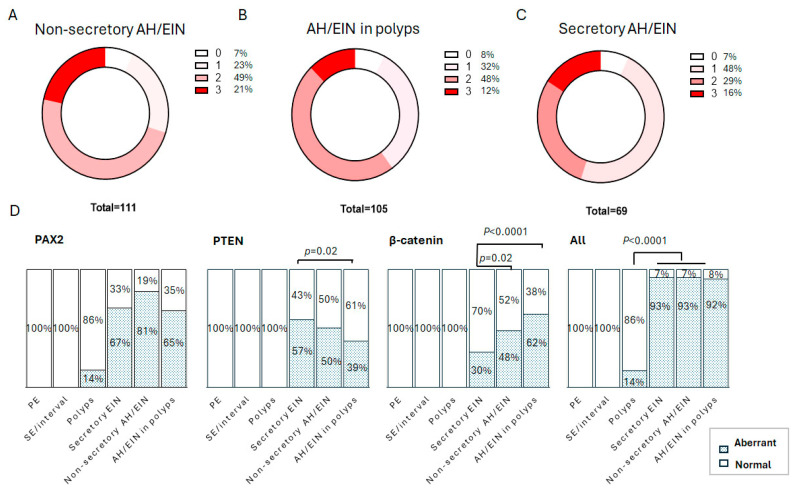
Comparison of 3-marker expression within various scenarios. (**A**) Performance of 3-marker panel in non-secretory bona fide AH/EIN. (**B**) Performance of 3-marker panel in AH/EIN in polyps. (**C**) Performance of 3-marker panel in secretory AH/EIN. (**D**) Comparison of aberrant expression of individual markers and any marker between proliferative endometrium (PE), secretory endometrium (SE) or interval endometrium (interval), secretory AH/EIN, non-secretory AH/EIN, and AH/EIN within endometrial polyps. *p*-value per Fisher’s exact test. Lines show significant differences between scenarios. Data drawn from previous study: PE (*n* = 79), polyps (*n* = 90), AH/EIN in polyps (*n* = 105), and non-secretory AH/EIN (*n* = 111).

In total, the sensitivity of the 3-marker panel in secretory AH/EIN was on a par with the sensitivity of previously published non-secretory bona fide AH/EIN [9] and AH/EIN in polyps [21], with around 93% of cases in all scenarios having one aberrantly expressed marker (Figure 6). The prevalence of PTEN aberrancy was significantly higher in secretory AH/EIN than in AH/EIN in endometrial polyps (*p* = 0.02). The prevalence of β-catenin aberrancy was significantly lower in secretory AH/EIN than in non-secretory bona fide AH/EIN and in AH/EIN within endometrial polyps (*p* = 0.02 and *p* = 0.0001). Data for non-secretory AH/EIN and AH/EIN within endometrial polyps were derived from previous studies [9,21].

## 4. Discussion

As detection of AH/EIN in secretory phase endometrium remains a challenging area in daily gynecologic pathology practice, the main goals of this study were to investigate the expression characteristics of the 3-marker panel within interval and secretory endometrium to refine interpretation criteria and determine the diagnostic sensitivity and specificity of the panel in identifying secretory AH/EIN. To our knowledge, no studies have specifically investigated AH/EIN in interval and secretory endometrium.

The expression characteristics of PAX2 and PTEN in interval endometrium and secretory endometrium were similar to those of proliferative endometrium [9] and endometrial polyps [21]. Previous studies of AH/EIN showed that focal sporadic loss of PAX2 and/or PTEN is common within benign endometrium [9,10,21,24]. As a result, PAX2 and PTEN interpretation criteria were established to have a 5% loss as a cutoff for aberrant expression. In this study, our findings confirm that sporadic loss of PAX2 and PTEN in secretory endometrium and interval endometrium is not uncommon. Additionally, we discovered that in less than 1% (*n* = 1) of cases of benign secretory endometrium, there was a 10% loss of PTEN. As previously established criteria were based on a relatively small sample size of benign endometrium cohort, it may be reasonable to adjust the cutoff criteria to 10%. It is worth further emphasizing that the aberrant expression of PAX2 and PTEN (≥10% loss) should not be confused with the sporadic, focal loss (<10% of the specimen) observed in benign endometrium. When interpreted in the context of morphologic findings, substantial loss of PAX2 or PTEN remains highly specific for AH/EIN. Therefore, IHC results should always be assessed alongside morphology, as IHC alone is not sufficient for diagnosis.

We also found that weak nuclear staining of β-catenin was prevalent in interval endometrium (defined as days 15–16 of the endometrial cycle and morphologically characterized as late proliferative endometrium with scattered subnuclear vacuoles), with weak nuclear blush present in 67% (*n* = 38) of interval endometrium cases, while it was completely absent in secretory endometrium. This phenomenon appears to be phase-specific rather than mutation-driven. The exact mechanisms underlying this weak nuclear staining remain unclear and warrant further investigation. Regardless of the cause, it is essential to refine the interpretation criteria for β-catenin aberrancy, defining it as strong, unequivocal nuclear staining rather than any nuclear staining. Failure to account for this distinction could lead to a potential diagnostic pitfall when evaluating interval endometrium. The 3-marker panel has now been tested across all phases of the menstrual cycle and in benign endometrial polyps. With the exception of frequent PAX2 aberrancy in endometrial polyps [21] and weak nuclear β-catenin expression in interval endometrium, the expression of all three markers in benign endometrium has remained consistent.

The 3-marker panel of PAX2, PTEN, and β-catenin effectively identified AH/EIN within interval and secretory endometrium with a high sensitivity of 93%. This performance was comparable to its accuracy in detecting non-secretory bona fide AH/EIN (92.8%) and AH/EIN within endometrial polyps (92.4%). Furthermore, the aberrant expression of the three markers showed no correlation with one another, indicating their independence. This independence enhances diagnostic confidence, as the presence of multiple marker aberrancy strengthens the diagnosis. This lack of association was not surprising, since all three markers came from separate pathways.

The prevalence of PAX2 and PTEN aberrancy in secretory AH/EIN was comparable to that reported in previous studies of non-secretory AH/EIN and AH/EIN within endometrial polyps. An interesting observation was that β-catenin aberrancy was significantly lower in secretory AH/EIN compared to previous studies of non-secretory AH/EIN and endometrial polyps. A possible explanation for this could be that the AH/EIN with mutated *CTNNB1* (β-catenin) is less responsive to progesterone, leading to fewer secretory changes.

One limitation of the 3-marker panel is its sensitivity ceiling (approximately 93%), underscoring the need for additional diagnostically useful biomarkers. An ideal immunohistochemical marker should meet the following criteria: (1) Established molecular basis—TCGA data identifying common mutations in key cell signaling pathways provide a strong foundation for selecting potential candidates. (2) Observable protein expression changes—genetic alterations should lead to detectable protein expression changes, preferably as a clear on/off switch or a distinct localization shift to minimize subjectivity. For instance, although PI3K and KRAS mutations frequently occur in AH/EIN and endometrioid carcinoma [6], they do not lead to protein expression changes, making them unsuitable as immunohistochemical markers; (3) Availability of commercial antibodies—for practical application, preferred markers should have commercially available antibodies. Without a readily available supply, a marker’s clinical utility is limited; (4) High prevalence in precancerous lesions—a substantial proportion of AH/EIN cases should exhibit aberrant marker expression. Although ARID1A, p53, and mismatch repair (MMR) proteins have high specificity for diagnosing AH/EIN [9], they are present in fewer than 10% of cases and do not improve the overall diagnostic sensitivity of the 3-marker panel, making them less valuable; (5) Minimal expression aberrancy in benign tissue—a marker should not display significant expression changes in benign counterparts. Additionally, our study investigated marker expression patterns in moderate-sized cohorts of benign endometrium and AH/EIN. Further validation in larger cohorts is necessary to strengthen these conclusions.

It is also worth noting that the 3-marker panel is an immunohistochemistry (IHC) test performed directly on tissue sections obtained through procedures such as random endometrial biopsy, hysteroscopy-guided biopsy, or dilation and curettage under general anesthesia. While this approach remains a valuable diagnostic tool, alternative methods have also shown utility in aiding the diagnosis of atypical hyperplasia/endometrial intraepithelial neoplasia (AH/EIN) and endometrial carcinoma. Recently, a novel biomarker known as SIR-EN—which combines the Systemic Inflammatory Index (SII) and endometrial thickness—has shown promise in predicting the risk of endometrial carcinoma in patients with abnormal uterine bleeding at menopause [25]. Additionally, circulating cell-free DNA (cfDNA) has emerged as a promising blood-based biomarker for endometrial cancer detection and monitoring [26,27,28,29,30]. These findings underscore the significant clinical potential of alternative markers as complementary tools in endometrial cancer risk assessment.

## 5. Conclusions

In conclusion, the three-marker panel comprising PAX2, PTEN, and β-catenin demonstrates potential in facilitating the identification of secretory AH/EIN, particularly in diagnostically challenging cases. Further studies are necessary to validate these findings and assess their clinical utility.

## Figures and Tables

**Table 1:** Summary of immunoprofiles of secretory AH/EIN.

Markers	Results	No/Mild Nuclear Atypia	With Nuclear Atypia	Total
PAX2	Intact	19 (34%)	4 (31%)	23 (33%)
	Loss	37 (66%)	9 (69%)	46 (67%)
PTEN	Intact	26 (46%)	4 (31%)	30 (43%)
	Loss	30 (54%)	9 (69%)	39 (57%)
β-catenin	Membranous	38 (68%)	10 (77%)	48 (70%)
	Nuclear	18 (32%)	3 (23%)	21 (30%)
Aberrant	All 3	9 (16%)	2 (15%)	11 (16%)
	PAX2 + PTEN	9 (16%)	4 (31%)	13 (19%)
	PAX2 + β-catenin	5 (9%)	1 (8%)	6 (9%)
	PTEN + β-catenin	1 (2%)	0 (0%)	1 (1%)
All normal		4 (7%)	1 (8%)	5 (7%)
Total		56	13	69

## Data Availability

Data can be made available by request.
